# 

**Published:** 1939

**Authors:** 


### The Medical Library of the University of Bristol

WITH WHICH IS INCORPORATED

**The Library of the Bristol Medico-Chirurgical Society.**

*The following donations have been received since the publication of the list in June,* 1939.

October, 1939.

| | | |
|---|---|---|
| Messrs. J. M. Dent & Sons, Ltd. (1) | .. .. | 1 volume. |
| Professor E. W. Hey Groves (2) | .. .. | 1 ,, |
| Hunterian Society (3) | .. .. .. .. | 1 ,, |
| Michell Clarke Fund (4) | .. .. .. .. | 1 ,, |
| Dr. W. Baly Peacock (5) | .. .. .. | 3 volumes. |
| Professor C. Bruce Perry (6) | .. .. .. | 3 ,, |

Unbound periodicals have also been received from Professor E. W. Hey Groves, Dr. T. Milling, Professor C. Bruce Perry, Dr. G. de M. Rudolf, and Dr. J. Odery Symes.

### THE ONE HUNDRED AND SIXTY-EIGHTH LIST OF BOOKS.

The figures in round brackets refer to the figures after the names of the donors. The books to which no such figures have been attached have either been bought from the Library Fund or received through the *Journal*.

| | | | |
|---|---|---|---|
| **Bach, F.** | *The Rheumatic Diseases* | | 1935 |
| **Barrington-Ward, Sir L.** (ed.) | *Royal Northern Operative Surgery* | | 1939 |
| **Beaumont, G.E., and Dodds, E.C.** | *Recent Advances in Medicine.* | 9th Ed. | 1939 |
| **British Medical Association.** | *Nutrition and the Public Health* | | 1939 |
| *Catalogue of Lewis's Medical and Scientific Library.* | | New Ed. Part II. | 1939 |
| **Coke, F., and Coke, H.** | *Asthma* | 2nd Ed. | 1939 |
| **Crowe, H. W.** | *Rheumatism* | | 1939 |
| **Cumberbatch, E. P.** | *Diathermy* | 3rd Ed. | 1937 |
| **Drinker, C. K.** | *Carbon Monoxide Asphyxia* | | 1938 |
| **Drummond, J. C., and Wilbraham, A.** | *The Englishman's Food* | | 1939 |

**Dukes, C. E.** .. .. *Urine Examination and Clinical Interpretation* 1939
**Evans, C. Lovatt** .. *Recent Advances in Physiology* .. 6th Ed. 1939
**Gasser, H. S., and others.** *Symposium on the Synapse* .. .. .. 1939
**Gordon, R. G.** (ed.) *Survey of Child Psychiatry* .. .. .. .. 1939
**Gould.** .. .. .. *Pocket Medical Dictionary* .. .. 11th Ed. 1939
**Harris, I.** .. .. .. *Diet and High Blood Pressure* .. .. .. 1937
**Hill, T. Rowland** (ed.) *Treatment of Some Common Diseases* .. .. 1939
**Hindley-Smith, J. D.** *The Streptococcal Tendency* .. .. .. .. 1939
**Kayne, G. G., and others.** *Pulmonary Tuberculosis* .. .. .. .. 1939
**Marshall, C. M.** .. *Cæsarean Section Lower Segment Operation* 1939
**Mitchiner, P. H., and Cowell, E. M.,** *Medical Organization and Surgical Practice in Air Raids* .. .. .. (2) 1939
**Mitton, R. G.** .. .. *Heat* .. .. .. .. .. .. .. .. .. .. .. (1) 1939
**Morris, H.** .. .. .. *Physiotherapy in Medical Practice* .. .. 1939
**Oriás, O., and Braun-Menéndez, E.** *Heart Sounds in Normal and Pathological Conditions* .. .. .. .. .. 1939
**Pearson, K.** .. .. *Treasury of Human Inheritance.* Vol. III, Part I. Vol. IV, Parts I–II .. .. 1925–35
**Pearson, W. J., and Watkins, A. G.** *The Infant* .. .. 2nd Ed. 1939
**Ride, L.** .. .. *Genetics and the Clinician* .. .. .. .. 1938
**Rorie, D.** .. .. .. *The Book cf Aberdeen* .. .. .. .. .. (6) 1939
**Royal Liverpool United Hospital.** *Hospital Testament* .. .. .. 1939
**Scott, G. R.** .. .. *The Story of Baths and Bathing* .. .. .. 1939
**Segal, J.** .. .. .. *Synopsis of Pulmonary Tuberculosis* .. .. 1939
**Smyth, J. C.** .. .. (i) *A Description of Jail Distemper;* (ii) *An Account of the Experiment made on board the Union Hospital Ship* (in one volume) 1795–96
**Spalteholz, W.** .. .. *A Hand Atlas of Human Anatomy.* 3 vols. 4th Ed. (5) 1923
**Stoll, A.** .. .. .. *The Cardiac Glycosides* .. .. .. .. .. (6) 1937
*Trabajos de la Càtedra de Semiologia. Secunda Serie* .. .. (6) 1935
**Treves, F.** .. .. *Surgical Applied Anatomy* .. .. 10th Ed. 1939
**Wakeley, C. P. G.** (ed.) *Modern Treatment in General Practice.* Vol. III .. 1937
**Widdowson, T. W., and Widdowson, E. V. B.** *Dental Surgery and Pathology* .. .. .. .. .. 3rd Ed. (4) 1937

TRANSACTIONS, REPORTS, JOURNALS, ETC.

Transactions of the Hunterian Society. Vol. III .. .. (3) 1938–39
Transactions of the Medical Society of London. Vol. LXII .. .. 1939

### Publications Received

From ACTINIC PRESS LTD.:

*Radiotherapy in Sinusitis.* By W. ANNANDALE JONES, M.C., M.D., Ch.B.

From THE BRITISH MEDICAL ASSOCIATION:

*Nutrition and the Public Health: Proceedings of a National Conference on the Wider Aspects of Nutrition, April 27th, 28th, 29th,* 1939. Price 2s. 6d.

From MESSRS. CASSELL & CO. LTD. :

*Claude Bernard, Physiologist.* By J. M. D. OLMSTEAD, M.A., Ph.D. Price 15s.

*Rectal Surgery.* By ERNEST MILES, F.R.C.S. Price 17s. 6d.

From H. K. LEWIS & CO. LTD. :

*Infections of the Hand.* By LIONEL R. FIFIELD, F.R.C.S. 2nd Edition by PATRICK CLARKSON, F.R.C.S. Price 9s.

*Sanitary Inspector's Handbook.* By HENRY H. CLAY, F.R.San.I., F.I.S.E. Price 17s. 6d.

*Common Skin Diseases.* 5th Ed. By A. C. ROXBURGH, M.A., M.D., B.Ch., F.R.C.P. Price 15s.

From UNIVERSITY PRESS OF LIVERPOOL :

*Hospital Testament. Issued by the Committee of the Royal Liverpool United Hospital.* Price 6d.

From MESSRS. E. AND S. LIVINGSTONE :

*Treatment of Some Common Diseases (Medical and Surgical).* By various authors. Edited by T. ROWLAND HILL, M.D., M.R.C.P. Price 15s.

*Textbook of Medical Treatment by Various Authors.* Edited by D. M. DUNLOP, B.A., M.D., F.R.C.P., L. S. P. DAVIDSON, B.A., M.D., F.R.C.P., M.R.C.P. and J. W. MCNEE, D.S.O., D.Sc., M.D., F.R.C.P. Price 25s.

From OXFORD UNIVERSITY PRESS :

*Thomson and Miles' Manual of Surgery.* Ed. 9. By ALEXANDER MILES, M.D., LL.D., F.R.C.S. and the late Sir DAVID WILKIE. Price 42s.

*Electric Excitation of Nerve.* By BERNHARD KATZ, M.D. (Leipzig), PH.D. Price 10s. 6d.

*Blood Transfusion.* By VICTOR HORSLEY RIDDELL, M.A., M.D. (Camb.), F.R.C.S. Price 21s.

*Urine-Examination and Clinical Interpretation.* By C. E. DUKES, M.Sc., M.D., D.P.H. Price 25s.

*A Survey of Child Psychiatry.* Contributed by contemporary British authorities. Edited on behalf of the Child Guidance Council by R. G. GORDON, M.D., D.Sc., F.R.C.P. Price 10s. 6d.

*Human Gastric Secretion.* By BENGT. J. E. IHRE, M.D. Price 12s. 6d.

*Heparin.* By J. ERIK JORPES, M.D. Price 7s. 6d.

*Pioneers in Acute Abdominal Surgery.* By ZACHARY COPE, B.A., M.D., M.S., F.R.C.S. Price 7s. 6d.

*Physics for Medical Students.* 2nd Ed. By J. S. ROGERS, B.A., M.Sc. Price 12s. 6d.

*Health in Relation to Occupation.* By H. M. VERNON, M.A., M.D., Price 15s.

From CHARLES C. THOMAS :

*Symposium on the Synapse.* By HERBERT S. GASSER and others.

From JOHN WRIGHT & SONS LTD. :

*Surgical Diagnosis.* By STEPHEN POWER, M.S., F.R.C.S. Price 12s. 6d.

*Abdominal Injuries of Warfare.* By GORDON GORDON-TAYLOR, O.B.E., M.A., F.R.C.S., F.R.A.C.S. Price 10s. 6d.

*British Journal of Surgery.* Vol. XXVII. No. 105 (July, 1939). Price 12s. 6d.

*Electrocardiograms.* By H. WALLACE JONES, M.D., M.Sc., F.R.C.P., and E. NOBLE CHAMBERLAIN, M.D., M.Sc., F.R.C.P. Price 3s. 6d.

*Diverticula and Diverticulitis of the Intestine : Their Pathology, Diagnosis and Treatment.* By HAROLD C. EDWARDS, M.S., F.R.C.S. Price 25s.

*Cæsarean Section : Lower Segment Operation.* By C. McINTOSH MARSHALL, F.R.C.S. Price 21s.

### Local Medical Notes

APPOINTMENTS.

The following appointments have been made in the **University of Bristol**:—

Dr. E. G. Bradbeer, Lecturer in Anæsthesia.
Dr. J. S. Baxter, Lecturer in Anatomy.
Dr. Norman Wood, Lecturer in Pathology.

EXAMINATION RESULTS.

**University of Bristol.**—Students of the University have recently passed the following examinations :—

M.B., Ch.B.—*Supplementary First Examination. Pass :* W. F. T. Baptiste, R. W. E. Brain, Roma N. Chamberlain, Jean R. Christie, Joan M. Doherty, Mary H. D. Douglas, W. A. Heaton-Ward, A. S. Jones, F. D. Kay, P. R. Miller, A. S. Nethercott, Ruth M. Rocke, Mary Waterhouse, W. P. Foster.

B.D.S.—*Supplementary First Examination. Pass :* C. R. W. Birkett.

L.D.S.—*Supplementary First Examination. Pass :* R. C. Taylor, F. E. R. Williams.

**Conjoint Board.**—L.R.C.P., M.R.C.S.—*Final Examination. Pass : In Medicine :* Barbara Cunningham,* Betty Fox.* *In Medicine and Surgery :* F. R. Hurford,* M. B. Dworkis. *In Medicine and Midwifery :* Megan E. Jones. *In Pathology :* Joan M. Foss.

L.D.S., R.C.S. — *Final Examination.* (*Parts I and II*). *Pass :* R. J. Gething.

L.S.A.—*Final Examination. Surgery Pass :* E. A. Griffiths.

* Qualified.

